# Malicious traffic detection combined deep neural network with hierarchical attention mechanism

**DOI:** 10.1038/s41598-021-91805-z

**Published:** 2021-06-11

**Authors:** Xiaoyang Liu, Jiamiao Liu

**Affiliations:** School of Computer Science and Engineering, Chongqing University of Technology, Chongqing, 400054 China

**Keywords:** Computational science, Computer science

## Abstract

Given the gradual intensification of the current network security situation, malicious attack traffic is flooding the entire network environment, and the current malicious traffic detection model is insufficient in detection efficiency and detection performance. This paper proposes a data processing method that divides the flow data into data flow segments so that the model can improve the throughput per unit time to meet its detection efficiency. For this kind of data, a malicious traffic detection model with a hierarchical attention mechanism is also proposed and named HAGRU (Hierarchical Attention Gated Recurrent Unit). By fusing the feature information of the three hierarchies, the detection ability of the model is improved. An attention mechanism is introduced to focus on malicious flows in the data flow segment, which can reasonably utilize limited computing resources. Finally, compare the proposed model with the current state of the method on the datasets. The experimental results show that: the novel model performs well in different evaluation indicators (detection rate, false-positive rate, *F*-score), and it can improve the performance of category recognition with fewer samples when the data is unbalanced. At the same time, the training of the novel model on larger datasets will enhance the generalization ability and reduce the false alarm rate. The proposed model not only improves the performance of malicious traffic detection but also provides a new research method for improving the efficiency of model detection.

## Introduction

With the continuous development of computer networks, it is changing people’s way of life and work. But there are so many security threats, this case is getting worse. So people came up with cybersecurity, this includes unauthorized access, abuse, policies, and practices to modify or reject. Cybersecurity primarily includes the CIA (Confidentiality, Integrity, Availability) of its carriers' information. Any activity that attempts to undermine the CIA or circumvent established cybersecurity mechanisms can be considered a cyber-intrusion. At present, IDS (Intrusion Detection System) is generally adopted in the security field to detect security attacks. It is a device or software application that monitors a network or system for malicious activity or policy violations. It is common to report any intrusion or violation to an administrator or to use security information and event management systems for centralized collection. IDS typically checks all incoming and outgoing flows from a particular network to determine whether each flow has signs of intrusion. Well-designed IDS and its associated features can identify the characteristics of most intrusions and automatically respond to them by writing security logs or issuing warnings.

According to the technical classification of intrusion detection system^1^, it can be divided into two categories: abuse detection and anomaly detection. Abuse detection is also called rule-based intrusion detection. In abuse detection, the trace left by the intrusion process model in the observed system is the basis of the decision. So the implementation defines some illegal characteristic behavior based on empirical rules or expert knowledge, then the observation object is compared with it to make a judgment of whether there is such an illegal behavior system. At present, the network structure is becoming more and more complex, and the intrusion methods are also developing with the trend of diversification and complexity. It brings more challenges to the intrusion detection system. Since the rise of machine learning, many studies have developed intrusion detection technology with machine intelligence. For example, SVM (Support Vector Machine)^2^, ANN (Artificial Neural Network)^3^, XGBoost^4^, GA (Genetic Algorithm), and integrated learning have achieved good results in the field of intrusion detection. But the machine learning algorithm can only be used as a classifier, it has many limitations as the intrusion becomes more and more complex and diversified.

Therefore, better learning methods are needed, especially in the automatic extraction of invasion features. Due to the extensive research and application of deep learning, especially in natural language processing, image processing, and speech processing, and great success in weather forecasting. The model with deep learning has a highly nonlinear structure, it is the ability to work well with complex datasets. And the development of parallel computing, it brings a new opportunity for the promotion and application of deep learning algorithm. As for the difficulty of training and computational complexity, RNN (Recurrent Neural Network) has failed to become a mainstream network model in the past few years. Nowadays, RNN has been successfully applied to handwriting recognition, speech recognition^5,6^, and machine translation^7^. The main feature of RNN is that it loops information in a hidden layer that remembers information that has been processed before, thus brings the structural advantage for processing the time-series information. Similarly, many network intrusion behaviors can be abstracted as events from a specific time series of the underlying network hierarchy model. Therefore, RNN is considered suitable for constructing the malicious traffic detection model in IDS.

Since Denning^8^ put forward the first intrusion detection model, academic researchers began to propose a variety of methods for intrusion detection. The purpose of intrusion detection is mainly to detect the traffic generated by attacks and illegal operations from the network. Malicious traffic is also the object of intrusion detection. First of all, the traditional malicious traffic detection method is mainly from statistics, classification, clustering, information theory four aspects to summarize the abnormal flow detection technology. Ahmed^4^ introduces various abnormal flow detection technologies using data mining, mainly from correlation analysis, clustering, classification, and the combination of a variety of data mining algorithms to conclude. Fernandes et al*.*^9^ summarized the current mainstream abnormal flow detection technology and the key issues and techniques in the detection process. Hande et al*.*^10^ introduced the methods and technical types of intrusion detection from three aspects of intrusion detection methods. However, traditional traffic detection methods cannot meet the security requirements of high performance. Then the rise of machine learning, its detection ability to obtain relatively high accuracy.

Then the rise of machine learning, its detection ability to obtain relatively high accuracy. Literature^11^ used a decision tree to realize abnormal flow detection, and used Lincoln laboratory data as training and test data. The unknown tag data is classified from top to bottom from the root node and tested by the root node or internal node, then select which branch to the next node based on the value of the test until the leaf is reached and the exception type is determined. Literature^12^ uses the C5.0 algorithm and C5.0 combined with other machine learning algorithms to improve the computational speed of the model. The utilization of memory resources and training times are reduced, so the efficiency and accuracy of the anomaly detection system are improved. At the same time, the wavelet transform and particle swarm optimization^13^ algorithm are used to optimize and change the decision tree model, to improve the performance of the model. They used naive Bayes to implement the anomaly detection technology and regarding the existence of the traffic characteristic attributes as an independent. The classification is judged according to the posterior probability maximization, and a good result is obtained. For example, they proposed a hybrid learning method. Mix k-means clustering, KNN, SVM, and naive Bayesian classification, and classify the results after clustering. The binary accuracy can be significantly improved to around 99.6% to 99.8% in the KDD dataset while reducing false alarms to 0.5%.

Finally, the rise of artificial intelligence, which uses neural networks to detect malicious traffic. Rahul Ahsan^14^ conducted a comparative experiment on the Shallow network and the deep neural network. It was found that the deeper network was more accurate to detect malicious traffic than the shallow neural network. Meanwhile, some researchers use CNN (Convolutional Neural Network) as feature extraction. Yu^13^ realized a semi-supervised detection method by combining CNN with a clustering method. More than 95% of the multi-classification accuracy was achieved on the dataset of network session based on a real network environment. Kim^15^ tested the Recurrent Neural Network (RNN) in the KDD99 dataset (Long Short Term Memory, LSTM) structure, which proves the strong ability of RNN to solve intrusion detection problems, but the input mode is not as convenient as CNN. Chawla^16^ applied the recursive neural network with a similar structure of RNN to the detection of the network security situation. The method extracts five-tuple and payload as input and introduces batch gradient descent to update model parameters. The prediction accuracy of the network security situation is greatly improved. Salman^17^ proposed a framework specifically for IoT device identification and malicious traffic detection. The framework pushes intelligence to the edge of the network and can extract functions by network flow to identify the source, the type of traffic generated, and detect network attacks. Nie^18^ proposed a mechanism based on reinforcement learning. We model the network traffic prediction problem as a Markov decision process and then use Monte Carlo Q learning to predict the network traffic. Abdellah^19^ uses LSTM-deep learning to perform IoT traffic prediction in time series. The prediction accuracy has been evaluated by using RMSE as the evaluation function and mean absolute percentage error (MAPE). Li^20^ proposed a deep learning method for intrusion detection using the multi-CNN fusion method. The experimental results successfully proved that the multi-CNN fusion model is very suitable for providing a classification method with high accuracy and low complexity on the NSL-KDD data set.

Current researches on malicious traffic detection are mainly based on a single data flow to determine whether it is malicious traffic, ignoring the correlation between a data flow and its immediate neighbor in a real network environment. At the same time, the data flow is detected one by one, reducing the efficiency of model checking. And the current detection method for detecting malicious traffic class imbalance data samples exist deficiencies in small sample categories. In order to improve the performance of malicious traffic detection in IDS, meanwhile to improve the data handling capacity of the model. This paper proposed a data processing method for data flow segmentation and a hierarchical attention model to detect different types of malicious traffic in the network. The proposed hierarchical attention model has a bi-directional GRU (Gated Recurrent Unit), then the feature fusion of three levels is composed of attention layer, maximum pooling layer, and average pooling layer, and finally, estimate whether the data flow is Malicious traffic.

### Our contributions

- A network malicious traffic detection model is proposed. A new malicious traffic detection structure is constructed combined deep neural network and hierarchical attention mechanical, then a detection algorithm is proposed. The novel model mainly uses the gated recurrent unit as the main memory unit and uses the attention mechanism layer, the three levels of maximum pooling layer and average pooling layer can extract rich flow characteristics. Finally, the types of malicious traffic are classified by using multi-layer perceptron units to provide security personnel analysis.
- In order to make the experiment close to the real detection environment, this paper also considers the impact of the imbalance of data samples. This paper proposes a data processing method of data flow segmentation, which can improve the throughput of the model and increase the detection efficiency of the model by stacking the data flow and dividing it into data segments.
- Detailed and systematic assessment and analysis are conducted on the three different datasets (NSL-KDD, CIC-IDS2017, and CSE-CIC-IDS2018). These three datasets have been widely used in advanced models, which makes the experimental results have a better comparison with the state of the art models. The proposed model is compared with six classical models. The experimental results show that the proposed HAGRU model has an *F*-score value of 96.71% and a detection rate (DR) value of 96.32% in intrusion detection. It is found that the malicious traffic detection model with an attention mechanism can recognize the aggressive traffic well.

## Dataset description

### NSL-KDD dataset

NSL-KDD dataset^21^ is widely used in intrusion detection experiments. In some network security intrusion detection experiments, almost all researchers use NSL-KDD as the benchmark dataset. NSL-KDD not only solves the problem of redundant samples inherent in the KDD Cup 1999 dataset effectively. Besides, the proportion of various samples of the dataset is reasonably adjusted to make the dataset categories more balanced. In this way, the traffic classifier model will not be biased towards more frequent data sample categories. NSL-KDD dataset includes training set (KDDTrain^+^) and test connection (KDDTest^+^). They have normal traffic records for four different types of attack traffic. As can be shown in Table 1, the statistical training set and test set contain the traffic data label categories of normal traffic and four types of attack traffic, respectively: Dos (denial of service attack), R2L (unauthorized access from a remote machine), U2R (unauthorized access to local super-user(root) privileges), and Probe (surveillance and other probin). After each traffic is numerically characterized, the eigenvector of traffic is obtained. There are a total of 41 features, including basic features, content features, and communication features. And there are some unique attack types in the test set, but the training set doesn't appear, the model is better able to reflect the model's actual malicious traffic detection capability on this test set.

**Table 1:** Label classification in NSL-KDD dataset.

Class	Total	Normal	Dos	Probe	R2L	U2R
KDDTrain^+^	125,973	67,343	45,927	11,656	995	52
KDDTest^+^	22,544	9711	7458	2421	2754	200

### CIC-IDS2017 dataset

CIC-IDS2017 dataset^22^ contains the common attack traffic data, it is in the real background traffic (normal traffic) to launch a simulated hacker attack, and through the monitor to collect network data traffic. This dataset covers a very wide range of traffic. For example, it has a complete network topology, including modems, firewalls, switches, routers, and a variety of operating systems (Windows, Ubuntu, and Mac OS) and a variety of attacks, probably including web-based attacks, brute force cracking, DoS, DDoS, common penetration attacks, cardiac bleeding, botnet, network scanning. Besides, the data type of attack traffic is calibrated according to the attacks in each period, as shown in Table 2, the distribution of various attack samples in the dataset is displayed. Since the normal flow is more than the attack traffic sample. Therefore, data balancing is needed to ensure the model’s generalization ability. CIC-IDS2017 dataset and CSE-CIC-IDS2018 dataset are transformed the traffic data into numerical vector information by feature processing, and the traffic characteristics can reach 79 items. It is more than the number of NSL-KDD features, and it is easier to improve the accuracy of the malicious traffic detection model.

**Table 2:** Dataset attack type distribution in CIC-IDS2017 dataset.

Attack name	Frequency	Attack name	Frequency
SSH-Patator	5897	Web Attack and Brute Force	1507
FTP-Patator	7938	Web Attack and XSS	652
DoS slowloris	5796	Web Attack and Sql Injection	21
DoS GoldenEye	10,293	Infiltration	36
Heartbleed	11	Bot	1966
DoS Slowhttptest	5499	PortScan	158,930
DoS Hulk	231,073	DDoS	128,027

### CSE-CIC-IDS2018 dataset

CSE-CIC-IDS2018 dataset^23^ is created by the Canadian Cyber Security Research Institute (CIC) and Communications Security agency (CSE). It simulated the traffic data collected by malicious users who may launch network attacks under the real network communication environment. This dataset recorded 10 days of network traffic (which includes both legitimate and malicious traffic). The dataset collected attacks in seven different scenarios, these included brute force, Heartbleed, botnets, DoS, DDoS, Web attacks, and intra network penetration. The dataset contained the sample flow category label. Category tags are divided into traffic generated by normal network traffic and aggressive network activity. Data feature information contained network interaction information. Such as protocol name, period, source IP (Internet Protocol), target IP, source port, destination port, etc. Table 3 lists the specific types of attacks and the corresponding number of samples. CIC team logged raw data daily, including network traffic and event logs. In the process of feature extraction from the original data, the research team used CICFlowMeter-V4.0 to extract more than 80 network traffic characteristics. Finally, the data is saved as a CSV (Comma Separated Value) file to facilitate the study of machine learning methods.

**Table 3:** Sample statistics of different attack types in CSE-CIC-IDS2018 dataset.

Attack name	Frequency	Attack Name	Frequency
DDOS attack-HOIC	686,012	DoS attacks-SlowHTTPTest	139,890
DDoS attacks-LOIC-HTTP	576,191	DoS attacks-GoldenEye	41,508
DoS attacks-Hulk	461,912	DoS attacks-Slowloris	10,990
Bot	286,191	DDOS attack-LOIC-UDP	1730
FTP-BruteForce	193,360	Brute Force -Web	611
SSH-Bruteforce	187,589	Brute Force -XSS	230
Infilteration	161,934	SQL Injection	87

## Dataset preprocessing

### Data processing summarize

**Figure 1:**
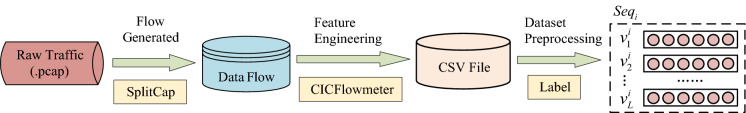
Traffic data processing.

The processing flow from the original flow data to the data input to the model is shown in Fig. 1. The first is to use the SplitCap^24^ tool to generate the data flow from the original captured traffic dataset (pcap file); then use the CICFlowmeter^25^ tool to do feature engineering on the data flow, and get the CSV format list processing result; finally, the CSV file data is subjected to data preprocessing (digitization, normalization, data missing value processing, data sampling, data flow segmentation) and data labeling. The result of the data flow segment obtained through the above-mentioned data processing flow is denoted by $Seq_{i}$, and its internal part is composed of $L$ data flow.

### Data sampling

**Figure 2:**
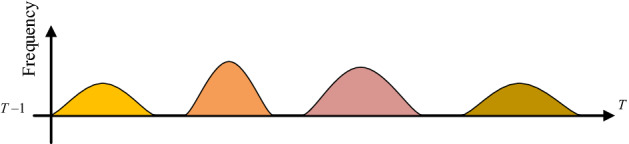
Network attack frequency diagram.

First, frequency sampling of malicious traffic. Through the analysis of network attack behavior, in general, a network attack is continuous in a period. If an attack is detected on the network, the corresponding attack traffic will frequently appear during this period. In order to simulate the training data closer to the frequency of attack requests in the real environment, local attack data is sampled. As shown in Fig. 2, it represents the attack frequency at different times during the period from $T{ - }1$ to $T$, and the peak waveforms of different colors represent different types of attacks. The attack frequency reflects the size of the attack traffic at this moment. When the attack frequency value is larger, the attack occurs more frequently in unit time; when the frequency value is 0, it means that there is no network attack traffic at the time, but only normal traffic.

Second, an Unbalanced data sample. Three datasets (NSL-KDD, CIC-IDS2017, and CSE-CIC-IDS2018) are used in the experiment. The number of sample categories of the CIC-IDS2017 and CSE-CIC-IDS2018 dataset are unbalanced. In the data category, there are more benign traffic than malicious traffic, and it is found in malicious traffic that different types of malicious traffic also have sample imbalances. Since the problem of data imbalance is a very common problem in deep learning, this paper adopts the method of data downsampling to alleviate the problem of sample imbalance.

### Data flow segmentation

**Figure 3:**
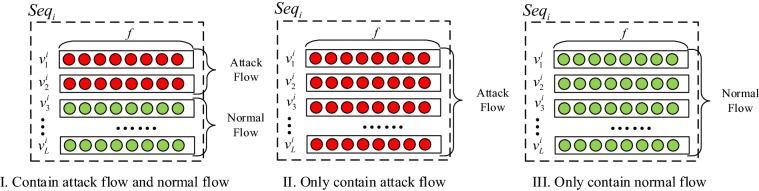
Data flow segment.

This paper performs frequency sampling and unbalanced sampling of the attack data flow, and then through the data processing of digitization, normalization, data missing value processing, the data $Seq_{i}$ ($i \in [1,B]$) of the input model is finally obtained. The structure is shown in Fig. 3 and $v_{1}^{i} ,v_{2}^{i} ,v_{3}^{i} ,...,v_{L}^{i}$ represents the preprocessed data flow. From the attack frequency in Fig. 2, we can see that when the length of the data flow segment $L$ is fixed, there are three situations in which $L$ flows are intercepted at a certain time from time segment $T - 1$ to $T$, as shown in Fig. 3 I, II, and III. It is that $L$ flows contain attack traffic and benign traffic, only attack traffic and only benign traffic, and mark the data flow segment.

In a real network environment, normal traffic is much larger than malicious traffic. By segmenting the data flow, it is obtained that most of the data flow segment is also benign traffic, and the rest is malicious traffic. The design purpose of the model is to allow benign traffic to pass through quickly, and only intercept malicious traffic, thereby increasing the throughput of the model and improving the detection efficiency of the model.

### Digitization

Three datasets are used in the experiment, only the NSL-KDD dataset requires numerical processing. The purpose is to convert character type features into numerical features. There are 38 numerical and 3 character features in the NSL-KDD dataset. Since the input of the malicious traffic detection model must be a numerical eigenvector, the non-numerical features must be numerically processed. Take the "protocol_type", "service", and "flag" features for example. The feature "protocol_type" has three properties, such as: "TCP", "UDP" and "ICMP", the characteristics of one-hot coding to (1, 0, 0), (0, 1, 0), (0, 0, 1) vector. As above, "service" has 70 attributes and "flag" has 11 attributes that require one-hot coding.

### Normalization

Three datasets are needed to do the data normalization. It can enable the parameter gradient to be updated in the correct direction each time, and also can reach the converge stably. For example, "duration [0,58329]", "src_bytes [0,1.3 × 109]", "dst_bytes [0,1.3 × 109]". There is a large difference between the maximum and minimum values of these eigenvalues, which requires the normalization of min–max and the linear transformation of the original data. The eigenvalues map between (0–1]. Numerical normalization is carried out by the method of min–max, as can be shown in the formula (1).

1 $$x_{i} = \frac{{x_{i} - Min}}{Max - Min}$$

### Data missing value processing

The characteristics of the traffic data extracted through the CICFlowMeter-V4.0 tool, there are missing values in a small number of samples. In this paper, the average method is adopted to deal with the characteristics of missing values. Other samples are used to carry out the weighted average of this feature, and then it is made up. Another case, which is different from the missing value, is that "NAN" and "Infinity" appear in the feature. This paper adopts the average method to fill it.

## Evaluation indicators

All possible results can be divided into the following four cases.

- True Positive (TP): actual attacks are classified as attacks.
- True Negative (TN): actual normal records are classified as normal.
- False Positive (FP): actual normal records are classified as attacks. This condition is also regarded as a false alarm.
- False Negative (FN): actual attacks are classified as normal records.

Then, the performance of the proposed model is evaluated by using different evaluation indicators:

2 $$Accuracy = \frac{TP + TN}{{TP + TN + FP + FN}}$$

Accuracy measures the proportion of the correctly classified traffic samples to the total traffic samples.

3 $$Precision = \frac{TP}{{TP + FP}}$$

Precision is to measure the malicious traffic detection model and predict the malicious traffic samples labeled as malicious in the proportion of the total malicious traffic samples.

4 $$Detection \, \;Rate\;(DR) = \frac{TP}{{TP + FN}}$$

The detection rate is to measure the proportion of malicious traffic labeled as malicious traffic in the detected malicious traffic by the model, to measure the ability for detecting malicious traffic.

5 $$False\; Positive \;Rate{\kern 1pt} \;{\kern 1pt} (FPR) = \frac{FP}{{FP + TN}}$$

False-positive rate is a measure of the probability that normal traffic is classified as malicious traffic by the detection model.

6 $$F{\text{-score}} = (1 + \beta^{2} ) \cdot \frac{Precision \cdot DR}{{\beta^{2} \cdot Precison + DR}}$$

*F*-score is a comprehensive factor composed of the balance of two factors, the precision and detection rate, which is an effective measure to evaluate the effectiveness of a model comprehensive detection, where $\beta$ is weight factor. There are two calculation method of *F*-score, and this paper takes the ‘macro’ way to calculate the overall sample evaluation.

On the one hand, from the point of view of the model classifier, the precision and detection rates are a pair of contradictory indicators. Higher accuracy means fewer false positives, but a higher detection rate also means fewer false positives. For example, if more suspicious attacks are classified as attacks (in extreme cases all traffic is classified as attack traffic), the detection rate will increase, but the accuracy will be greatly reduced, and vice versa. Therefore, a single high precision or detection rate is not meaningful. On the other hand, from an intrusion detection perspective, especially in some strict environments (network environments require a high degree of security, especially in e-commerce and Banks network), the intrusion tolerance is very low, so the single detection rate is also an important indicator. *F*-score measure is a comprehensive consideration of the accuracy and detection rate, and the *F*-score is the harmonic average based on the precision and the detection rate. The higher the *F*-score, the higher the precision and detection rate.

## Methods

### Structure

**Figure 4:**
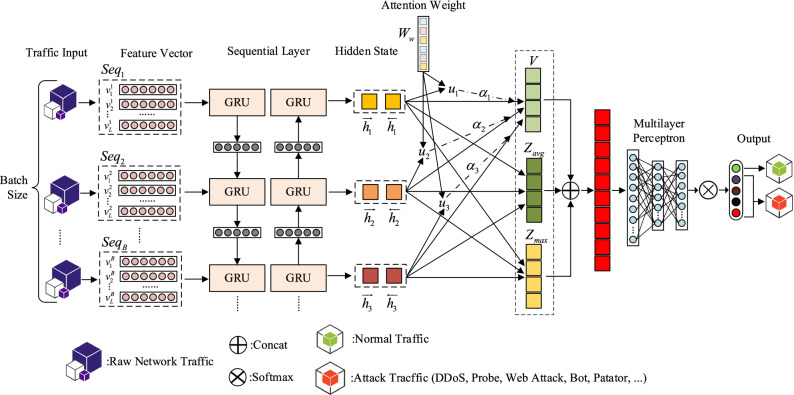
Proposed malicious traffic detection structure.

The data flow segment is obtained by data preprocessing. A combined deep neural network with a hierarchical attention mechanism, a novel network malicious traffic detection structure is proposed. The new model is based on the currently effective, reliable deep recurrent neural network. Compared with the traditional neural network for malicious traffic detection methods, it has high detection accuracy, low false alarm rate, and relatively good real-time performance. The structure is constructed in Fig. 4.

The proposed hierarchical attention model designed for malicious traffic detection is divided into five parts. Namely, the input layer, the feature conversion part, the bidirectional gated memory unit part, the hierarchy part, and the multi-layer perceptron output part. The "hierarchy" defined in the research of this paper means to perform further different operations on the hidden state *h* of the bidirectional gated recurrent neural network (BiGRU). According to the data flow segment obtained by data preprocessing, three different operations are performed on the hidden state information, namely the attention mechanism hierarchy, the maximum pooling hierarchy, and the average pooling hierarchy, and the attention mechanism hierarchy contains only one layer. The results of these three levels of operations are stacked to obtain richer traffic features, making it easier for the model to identify malicious traffic. The main function in the attention hierarchy is to focus on the recognition of malicious flow in the data flow segment, and the soft attention mechanism is used in the attention level of this paper, with only one attention weight *W*_*w*_. Therefore, it has the attention to the data flow level in the data flow segment. The maximum pooling hierarchy introduces abstract expressions to alleviate the over-fitting phenomenon during model training. The average pooling level can reduce the variance of the estimated value caused by the limited neighborhood size and improve the generalization ability of the model. Besides, both the maximum pooling hierarchy and the average pooling hierarchy can reduce model learning parameters and reduce the cost of model inference. Since the information extracted by the attention mechanism hierarchy in the HAGRU malicious traffic recognition model proposed in this paper is very critical, it is defined and described as a hierarchical attention mechanism.

### GRU (Gated Recurrent Unit)

**Figure 5:**
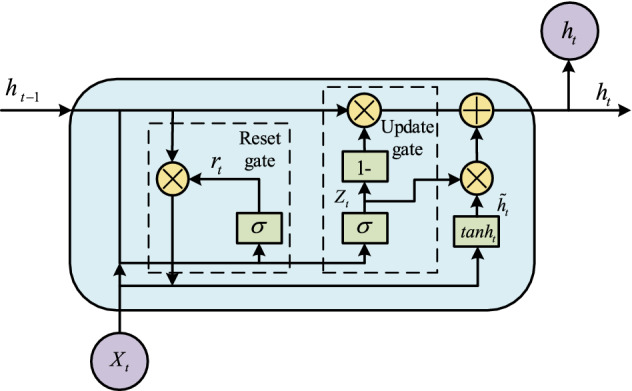
Structure of GRU.

GRU^26^ network is obtained according to the LSTM network variant. Compared with LSTM, GRU lacks a gate. Therefore, the number of parameters is less than LSTM. The traffic detection model has at least two characteristics: (1) the ability to minimize parameters; (2) the ability to process time-series data. So GRU is used as a part of the proposed model. According to Fig. 5, some specific inside structures of the GRU model is shown. GRU model is mainly represented by the update gate and the reset gate by $z_{t}$ and $r_{t}$ respectively. Compared with the LSTM model, there is one less gating signal, so the parameter number of GRU is decreasing.

The update gate $z_{t}$ is used to control the degree to which the previous state information is brought into the current state. The larger the value of the update gate, the more state information from the previous moment is entered.

7 $$z_{t} = \sigma (W_{z} x_{t} + U_{z} h_{t - 1} )$$

Reset gate $r_{t}$ controls the information from the previous state which is written to the current candidate set $\tilde{h}_{t}$, and the smaller the reset gate, the less information from the previous state is written to.

8 $$r_{t} = \sigma (W_{r} x_{t} + U_{r} h_{t - 1} )$$

where $x_{t}$ is plugged into the network unit, it is multiplied by its weight $W_{r}$. The same goes for $h_{t - 1}$ which holds the information for the previous $t - 1$ units and is multiplied by its weight $U_{r}$. Both results are added together and a Sigmoid activation function is applied to squash the result between 0 and 1.

Current memory content can be denoted by

9 $$\tilde{h}_{t} = tanh(W_{h} x_{t} + r_{t} \odot Uh_{t - 1} )$$

Multiply the input $x_{t}$ with weight $W_{h}$ and $h_{t - 1}$ with a weight $U$, then calculate the Hadamard product between the reset gate $r_{t}$ and $Uh_{t - 1}$.

Final memory at the current time step can be expressed by

10 $$h_{t} = (1 - z_{t} )h_{t - 1} + z_{t} \tilde{h}_{t}$$

Step 1: Apply element-wise multiplication to the $z_{t}$ and $h_{t - 1}$;

Step 2: Apply element-wise multiplication to $(1 - z_{t} )$ and $\tilde{h}_{t}$; then sum the results from Step 1 and Step 2.

### Traffic flow encoder

The bidirectional GRU model is used in the HAGRU model proposed in this paper. Since the GRU model is time-sequential, there are two sequences for feature extraction of traffic segments, which are from front to back, represented by $\overrightarrow {{h_{t} }}$, and from back to front, represented by $\overleftarrow {{h_{t} }}$; and finally merged into $h_{t}$.

11 $$\overrightarrow {{h_{t} }} = \overrightarrow {GRU} (Seq_{i} ),i \in [1,B],t \in [1,L]$$

12 $$\overleftarrow {{h_{t} }} = \overleftarrow {GRU} (Seq_{i} ),i \in [1,B],t \in [L,1]$$

13 $$h_{t} = \left[ {\overrightarrow {{h_{t} }} ,\overleftarrow {{h_{t} }} } \right]$$

### Activation functions

In the neural network, the activation function is mainly used to carry out a nonlinear transformation of the numerical value of the neural network unit. It can increase the nonlinearity of the neural network model and improve the expression ability of the neural network model. The hyperbolic tangent function can be represented by formula (14). An activation function can be used in the attentional mechanism. Formula (15) denotes ReLU (Rectified Linear Unit) of activation function in different layers.

14 $$f_{1} (x) = tanh(x)$$

15 $$f_{2} (x) = max(0,w^{T} x + b)$$

### Attentional mechanism

Traffic detection environments are typically deployed on firewalls, the hardware platform hosted by the firewall is usually limited in computing resources and storage resources, more than the rated bandwidth traffic makes the firewall become the bottleneck of the network transmission link, which is not conducive to the network transmission. Especially in the case of limited computing resources, more should make traffic through the firewall in real-time. Therefore, the traffic detector must use reasonable computer resources. And the attentional mechanism can exactly solve a difficult problem, attention mechanism is a resource allocation scheme that is the main means to solve the problem of information overload. The rational and effective utilization of computing resources enables the detection model to focus on the recognition of malicious traffic feature maps.

**Figure 6:**
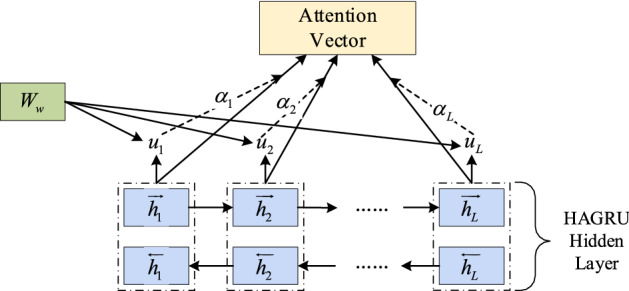
soft attention mechanism of HAGRU model.

The attention mechanism is divided into soft attention^27^, hard attention, and self-attention. This paper adopts the soft attention mechanism. First of all, the model has an attentional weight matrix that can be trained, after activating the function, the value is transferred to the Softmax function to obtain a weight value, and the $K$ dimension weight vector of the value sum is equal to 1. Finally, the attention vector can be obtained by weighted calculating of the hidden state. The schematic diagram of soft attention is shown in Fig. 6.

16 $$u_{t} = \tanh (W_{w} h_{t} + b_{w} )$$

17 $$\alpha_{t} = \frac{{\exp (u_{t}^{T} u_{w} )}}{{\sum\limits_{t} {\exp (u_{t}^{T} u_{w} )} }}$$

18 $$V = \sum\limits_{t} {\alpha_{t} h_{t} }$$

where $h_{{\text{t}}}$ represents the hidden state, $W_{w}$ denotes attentional weight matrix,$b_{w}$ is attention bias,$\alpha_{t}$ expresses the weight ratio matrix, $V$ indicates the attentional mechanism weighted attentional vector.

### MaxPooling and AvgPooling

In the malicious traffic detection model based on the hierarchical attention mechanism proposed in this paper, max-pooling and avg-pooling operations are used. The max-pooling is applied to the hidden layer $h$, and $C_{ij}$ is used to represent each feature mapping value of $h$. The one-dimensional max-pooling is used, the max-pooling is applied to the hidden layer $h$(shapes as $[l \times n]$), and $C_{i,j}$($0 \le i < l$,$0 \le j < n$)is used to represent each feature mapping value of $h$.Through formula (19), the max value $C_{i,M}$ of each dimension is calculated by taking the filter.

19 $$C_{i,M} = max(C_{i,0} ,C_{i,1} , \ldots ,C_{i,j} )$$

The hidden layer finally gets the one-dimensional vector $$Z_{\max } = [\begin{array}{*{20}c} {C_{1,M} } & {C_{2,M} } & {, \ldots ,} & {C_{l,M} } \\ \end{array} ]$$ through the max-pooling result.

And avg-pooling is similar to max-pooling, but the only difference is that when you calculate the value of this feature map in $h$, the average operation is used instead of selecting the max operation, and gets one-dimensional vector $Z_{avg}$.

### Multilayer perceptron

MLP (multilayer perceptron) is a feedforward neural network that maps a set of input vectors to output vectors. There is a nonlinear activation function element at each node. For example, formula (20) indicates that the calculation of a neural network is completed, it needs to pass the value to the next neuron by using the activation function (21).

20 $$D_{j} = f(\sum\limits_{k = 0}^{H} {W_{kj} \cdot x_{k} + b_{j} } )$$

21 $$D = [D_{1} ,D_{2} , \ldots ,D_{j} , \ldots ,D_{l} ]$$

where $W_{kj}$ represents the weight vector for $x_{k}$ in *j*-th dense unit,$b_{j}$ denotes the bias of *j*-th dense unit,$H$ indicates how many neural units are in the next layer, for each unit it can get output alias as $D_{j}$, finally the dense result $D$ can concatenate the output of each unit.

### Softmax for output

Softmax is a kind of logistic regression function. Under the label of $K$ class of dataset, the one-dimensional vector $\sigma (x)$ of $K$ dimension with the value of (0,1) is obtained. The vector formula can be denoted by.

22 $$\sigma (x)_{j} = \frac{{e^{{x_{j} }} }}{{\sum\nolimits_{k = 1}^{K} {e^{{x_{k} }} } }} \, j = 1, \ldots , K$$

The multi-classification task can be accomplished by using Softmax in the final phase of the traffic classification output. MLP should output $x$ to Softmax in order to build a multi-classifier, and a hypothesis function is needed to estimate the probability $P(y = j|x)$ of each class $j$. In other words, it needs to estimate the probability of the output of each possible category. Specifically, the hypothesis function should output a $K$-dimensional vector (the sum of the elements of the vector is 1) to represent the estimated probability. The hypothesis function can be expressed by

23 $$h_{\theta } (x^{(i)} ) = \left[ {\begin{array}{*{20}c} {P(y^{(i)} = 0|x^{(i)} ;\theta )} \\ {P(y^{(i)} = 1|x^{(i)} ;\theta )} \\ \vdots \\ {P(y^{(i)} = k - 1|x^{(i)} ;\theta )} \\ \end{array} } \right] = \frac{1}{{\sum\nolimits_{j = 0}^{k - 1} {e^{{\theta_{j}^{T} x^{(i)} }} } }}\left[ {\begin{array}{*{20}c} {e^{{\theta_{0}^{T} x^{(i)} }} } \\ {e^{{\theta_{1}^{T} x^{(i)} }} } \\ \vdots \\ {e^{{\theta_{k - 1}^{T} x^{(i)} }} } \\ \end{array} } \right]$$

where $h_{\theta } (x^{(i)} )$ is the hypothesis function, and $\theta_{0} ,\theta_{1} , \ldots ,\theta_{k - 1}$ is a fixed parameter, $1/\sum\nolimits_{j = 0}^{k - 1} {e^{{\theta_{j}^{T} x^{(i)} }} }$ is the normalization factor of hypothesis function. Furthermore, if $\theta \to \infty$, Softmax will become the maximum function. When taking different finite values, Softmax can be considered a parameterized and softened version of the maximization function.

### Loss of function

The cross-entropy loss function (objective function) is used to calculate the loss value between the true label and the predicted label, and the loss value is used. Take the derivative of backpropagation, the iteration of the gradient is updated, and finally, the approximate optimal solution $\theta$ can be obtained. Equation (24) is the expression of cross-entropy loss. It is suitable for the calculation of binary or multi-classification loss function.

24 $$J(\theta ) = - \left[ {\sum\limits_{i = 1}^{m} {y^{(i)} \log h_{\theta } (x^{(i)} ) + (1 - y^{(i)} )\log (1 - h_{\theta } (x^{(i)} ))} } \right]$$

where $m$ is the number of the training samples, the weight of the being trained is $\theta$. The train set is $\left\{ {\left( {x^{(1)} ,y^{(1)} } \right), \ldots ,(x^{(m)} ,y^{(m)} )} \right\}$, the training sample label has $K$ classes, so $y^{(i)} \in \left\{ {1,2, \ldots ,K} \right\}$.


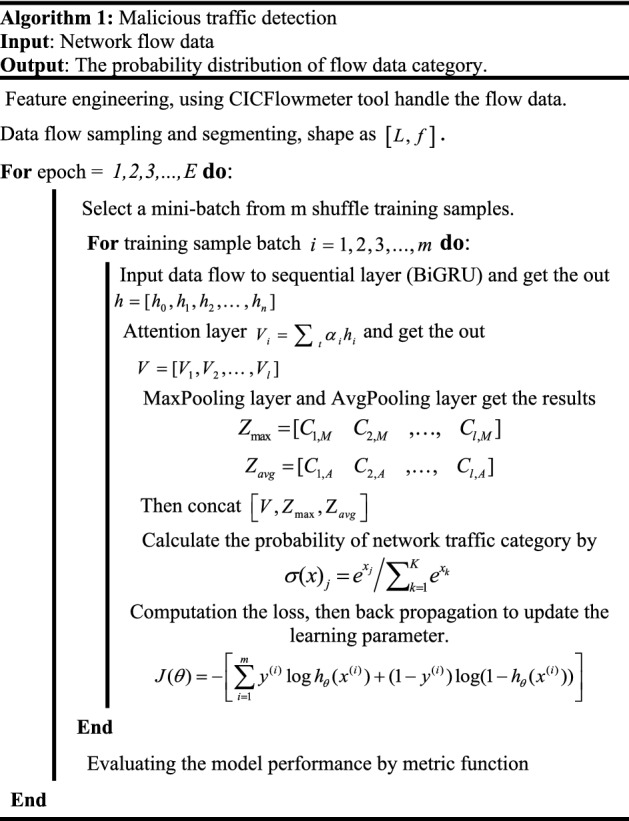


### Algorithm

The attention mechanism layer enables the model to keep the computational force constant to get more model performance improvements and better identification between malicious and normal traffic. This paper not only uses attention mechanism to extract important features but also uses the maximum pooling feature and average pooling feature for fusion. Rich feature information is extracted from the original feature map, which makes the model has a high detection accuracy. The multi-layer perceptron transforms the features of the hierarchical fusion linearly, and finally outputs the category of traffic. Proposed malicious traffic detection is shown in Algorithm 1.

## Results

Experiments are used to prove the feasibility of this model in three different datasets of NSL-KDD, CIC-IDS2017, and CES-CIC-IDS2018. In the experiment, data frequency sampling and data imbalance are processed, and the training set and test set are divided on each dataset, the ratio is 8:2.

The proposed model is evaluated by the detection rate and the false positive rate. We select the state of the art six methods for the comparison of malicious traffic detection, two of which are detection models based on machine learning, and the other four are traffic detection models based on deep learning, special as follows:

- **XGBoost**^28^: The model is biased towards classes with more samples, and because the features of the minority class are usually regarded as noise and therefore ignored, they also tend to predict the problem of only the majority class data. An integrated classification model of XGBoost combined with a tree method is proposed to improve classification performance.
- **SwiftIDS (LightGBM-based)**^29^: An intrusion detection system that can analyze a large amount of traffic data in a high-speed network in time and maintain satisfactory detection performance is proposed. And use a light gradient boosting machine (LightGBM) as an intrusion detection algorithm to process massive traffic data.
- **Deep Packet (CNN)**^30^: Design a Deep Packet framework for network traffic recognition, and embed an improved convolutional neural network in the framework as a traffic recognition model.
- **Deep Packet (SAE)**^30^: Design a Deep Packet framework for network traffic identification, and embed a stacked autoencoder in the framework as a traffic identification model.
- **Multi-CNN**^20^: Using multiple CNN models to predict the results and merging voting to determine the final prediction result.
- **Deep-Full-Range (Multi-LSTM)**^31^: A lightweight framework for traffic identification is proposed, and multi-layer LSTM is adopted as the traffic identification model.

The experimental results are the performance of the model on the test set in Tables 4, 5 and 6. The columns of Tables 4, 5 and 6 show the specific sample types in each dataset, the rows show the proposed model and the comparison models. DR, FPR and *F*-score are chosen as the main evaluation indicator. The number of evaluation indicators is between [0–1]. DR is higher, the better the model works on this kind of data. The same case is suitable for *F* -score. However, the lower of the FPR value, the better of the results. The smaller the false positives, the better in the detection of malicious traffic. It can improve network security, reduce the security problems caused by false positives.

**Table 4:** Performance comparison of each model in NSL-KDD dataset (units: %, bold indicates the best value).

Model name	XGBoost			SwiftIDS (LightGBM-based)			Deep packet (CNN)			Deep packet (SAE)			Multi-CNN			Deep-full-range (multi-LSTM)			Proposed model (HAGRU)		
Class	DR	FPR	*F*-score	DR	FPR	*F*-score	DR	FPR	*F*-score	DR	FPR	*F*-score	DR	FPR	*F*-score	DR	FPR	*F*-score	DR	FPR	*F*-score
Normal	99.76	0.814	99.50	99.30	0.807	99.27	98.89	0.884	99.03	99.13	1.232	98.99	98.97	0.800	99.11	99.41	0.772	99.34	99.47	0.765	99.37
DoS	99.95	0.074	99.91	99.87	0.095	99.85	99.79	0.206	99.71	99.82	0.185	99.75	99.82	0.179	99.75	99.84	0.058	99.87	99.88	0.074	99.87
Probe	98.47	0.059	98.94	98.87	0.148	98.71	98.22	0.233	97.97	96.33	0.207	97.12	98.72	0.196	98.40	98.80	0.111	98.85	98.47	0.100	98.74
R2L	92.71	0.034	95.54	90.41	0.207	91.09	90.95	0.300	89.74	89.47	0.245	89.89	90.95	0.293	89.86	91.49	0.207	91.68	91.76	0.186	92.20
U2R	63.79	0.004	77.08	77.58	0.003	86.53	81.03	0.006	87.85	77.58	0.016	83.33	81.03	0.006	87.85	77.58	0.013	84.11	81.04	0.006	87.86
Macro	90.93	–	94.19	93.21	–	95.09	93.78	–	94.86	92.46	–	93.81	93.90	–	94.99	93.42	–	94.77	**94.12**	–	**95.6**1

Table 4 shows the evaluation indicators of each model on the NSL-KDD dataset. For the convenience of observation, all values in the table are in hundredths. It indicates that the DR and F-score of the proposed HAGRU model in this paper are better than that of the compared models. HAGRU model does not superior to the compared models in terms of performance indicators in the categories of Normal, DoS, Probe, and R2L of NSL-KDD dataset, while it is better than the compared models in the last category U2R. In this way, the HAGRU model is better than the compared models in the overall sample evaluation index and achieved the DR of 94.12% and *F-*score is 95.61%. Due to the unbalanced data sample category on the NSL-KDD dataset, and even if the data has been sampled, this problem cannot be completely solved. Moreover, the data used in malicious traffic detection cannot be enhanced to expand data diversity. However, because the HAGRU model adopts the attentional mechanism, that is, it can make a good identification for the case with a small amount of data and samples. The performance of the proposed model in unbalanced datasets is better.

Table 5 shows the performance of each model on the CIC-IDS2017 dataset. In the experiment, CIC-IDS2017 is re-classified and sampled. Because the amount of original data categories (such as Web Attack & Brute, Web Attack & XSS, Web Attack & SQL) is too small, it is unable to meet the experimental requirements, the three categories are reclassified as Web Attack. Similarly, with other similar samples. According to the similarity of Attack types, Bot, DDoS, DoS, Patator, PortScan, and Web Attack are classified into six attack categories. The HAGRU model is also better than the compared models in terms of the performance of the total samples. DR and *F*-score are 96.32% and 96.71%, respectively, but it should also be noted that not every evaluation indicator is good in all categories. The proposed HAGRU model is better than other models from a comprehensive perspective, especially in the case that some categories are unbalanced. For example, the *F*-score of Web Attack category reaches 98.52%, which is higher than the *F*-score of other models. When the value of FPR is very low, even if the value of false alarm rate is very low, the performance of the HAGRU model is not necessarily good. We need to further examine the value of F-score, for example, using the Deep packet (SAE-based) model to classify the Bot. Although FPR is 0.020, but the *F* -score is75.21, which is smaller than other models. In this case, the performance of the Deep packet (SAE-based) model for Bot classification is very poor. Similarly, the performance of the model is considered to be poor whenever such a similar situation occurs in the model. The proposed HAGRU model has a certain improvement in the classification of CIC-IDS2017 data of different types of data flow compared with other models.

**Table 5:** Performance comparison of each model in CIC-IDS2017 dataset (units: %, bold indicates the best value).

Model name	XGBoost			SwiftIDS (LightGBM-based)			Deep packet (CNN-based)			Deep packet (SAE-based)			Multi-CNN			Deep-full-range (multi-LSTM)			Proposed model (HAGRU)		
Class	DR	FPR	*F*-score	DR	FPR	*F*-score	DR	FPR	*F*-score	DR	FPR	*F*-score	DR	FPR	*F*-score	DR	FPR	*F*-score	DR	FPR	*F*-score
Normal	98.61	0.129	99.17	99.50	0.249	99.50	98.64	0.069	99.24	98.99	0.174	99.31	98.87	0.189	99.24	99.27	0.209	99.42	99.37	0.160	99.52
Bot	89.05	0.077	80.18	67.68	0.022	76.32	94.65	0.068	84.64	66.41	0.020	75.21	96.18	0.108	79.41	68.70	0.019	77.69	76.33	0.035	79.68
DDoS	99.98	0.066	99.80	99.94	0.005	99.95	99.90	0.002	99.94	99.94	0.002	99.96	99.64	0.001	99.81	99.95	0.003	99.96	99.94	0.001	99.96
DoS	99.84	0.398	99.46	99.76	0.193	99.65	99.96	0.467	99.44	99.94	0.378	99.53	99.89	0.386	99.50	99.84	0.192	99.69	99.89	0.241	99.66
Patator	99.71	0.009	99.58	99.38	0.005	99.54	99.45	0.017	99.20	99.56	0.013	99.38	99.31	0.001	99.61	99.67	0.004	99.71	99.67	0.006	99.65
PortScan	99.92	0.021	99.91	99.95	0.0007	99.97	99.92	0.022	99.91	99.92	0.005	99.95	99.89	0.004	99.93	99.95	0.0007	99.97	99.92	0	99.96
Web Attack	97.48	0.038	91.90	97.25	0.010	96.70	95.88	0.046	89.72	96.10	0.048	89.55	91.07	0.013	92.77	97.71	0.088	84.38	99.08	0.005	98.52
Macro	97.80	–	95.71	94.78	–	95.95	**98.34**	–	96.01	94.41	–	94.70	97.84	–	95.75	95.01	–	94.40	96.32	–	**96.71**

Table 6 shows the performance of various models on the CSE-CIC-IDS2018 dataset. According to the statistics of each attack sample in the CSE-CIC-IDS2018 dataset, some types of attacks are very few, resulting in serious dataset balancing with other samples. So dada sample imbalance process should be made, at the same time, sample redefinition of labels is also needed. In this paper, the three types of composite Web attacks, namely Brute force-web, Brute force-xss, and SQL Injection, are synthesized according to the Attack approximate premise. Thus, there are 13 categories in the CSE-CIC-IDS2018 dataset: Benign, DDoS attacksloic-http, Bot, DDoS attack-hoic, DoS attack-hulk, ftp-brute Force, ssh-brute Force, Infilteration, DoS Attacks lowHTTPTest, DoS Attacks-GoldenEye, DoS Attacks-SlowLoris, DDoSAttack—LOIC—UDP, Web—Attack. The proposed HAGRU model in this paper can still achieve good results in the case of total samples. The values of DR and *F*-score are 93.06% and 93.95%, respectively. For each type of network malicious traffic attack, the proposed HAGRU model basically has some performance improvement compared with other models. Moreover, HAGRU model achieves 0 false alarm rate, DR and F-score close to 100% in the five categories of DDoS attack-HOIC,DDoS attack-HOIC,SSH-Brute Force, DoSAttacks-SlowLoris, DDoSAttack-LOIC-UDP. It shows that the proposed model can recognize this kind of attack very well.

**Table 6:** Performance comparison of each model in CES-CIC-IDS2018 dataset (units: %, bold indicates the best value).

Model name	XGBoost			SwiftIDS (LightGBM-based)			Deep packet (CNN)			Deep packet (SAE)			Multi-CNN			Deep-full-range (multi-LSTM)			Proposed model (HAGRU)		
Class	DR	FPR	*F*-score	DR	FPR	*F*-score	DR	FPR	*F*-score	DR	FPR	*F-*score	DR	FPR	*F*-score	DR	FPR	*F*-score	DR	FPR	*F*-score
Benign	97.70	3.70	88.79	98.60	2.759	91.60	96.25	4.345	86.55	96.97	4.426	86.70	96.14	4.694	85.69	95.07	3.733	87.40	98.37	1.617	94.52
DDoSattacskLOIC-HTTP	99.92	0.003	99.95	99.99	0.001	99.99	100	0.017	99.94	99.88	0.006	99.91	98.92	0.020	99.39	99.98	0.006	99.96	99.99	0.0003	99.99
Bot	99.98	0.004	99.98	99.99	0.001	99.99	99.90	0.002	99.94	99.97	0.004	99.97	99.91	0.003	99.94	99.98	0.004	99.98	99.99	0.001	99.99
DDoS attack-HOIC	100	0.04	99.87	100	0.006	99.97	100	0	99.99	100	0	100	100	0	100	100	0.0005	99.99	100	0	100
DoS Attack-Hulk	100	0.002	99.99	100	0.0005	99.99	100	0.029	99.86	99.99	0.384	98.19	99.91	0.711	96.67	99.82	0.036	99.73	100	0	100
FTP-Brute Force	88.48	3.54	79.23	96.33	3.486	83.53	89.48	3.703	79.11	87.86	3.589	78.73	87.62	3.754	77.98	89.00	3.601	79.21	96.34	3.487	83.53
SSH-Brute Force	99.98	0	99.99	99.98	0	99.99	99.98	0.001	99.98	99.98	0.0003	99.99	99.97	0	99.98	99.98	0.0005	99.99	99.98	0	99.99
Infilteration	58.51	3.28	72.02	69.01	0.208	80.46	51.68	0.563	65.23	50.40	0.461	64.62	49.18	0.593	62.90	58.40	0.752	69.76	81.88	0.247	88.57
DoSAttacks lowHTTPtest	51.67	1.14	61.64	52.41	0.363	66.54	49.40	1.031	60.28	50.99	1.198	60.77	48.36	1.218	58.40	50.80	1.078	61.22	52.39	0.361	66.54
DoSAttacks-GoldenEye	99.93	0.002	99.92	99.95	0.001	99.93	98.75	0.0009	99.35	82.19	0.0002	90.22	67.09	0.007	80.11	98.34	0.017	98.73	99.96	0.0007	99.96
DoSAttacks-SlowLoris	99.30	0.002	99.51	99.90	0.001	99.81	100	0.0020	99.79	100	0.002	99.79	99.95	0.001	99.86	99.95	0.002	99.77	100	0	99.93
DDoSAttack-LOIC-UDP	100	0.0002	99.85	100	0	100	100	0	100	100	0.0002	99.85	100	0	100	100	0	100	100	0	100
Web-Attack	52.60	0	68.93	75.14	0	85.80	69.85	0.001	81.11	76.30	0.004	81.98	80.00	0.001	87.19	75.59	0.002	83.81	80.92	0.001	88.32
Macro	88.31	–	89.97	91.64	–	92.89	88.87	–	90.09	88.04	–	89.28	86.69	–	88.32	89.76	–	90.73	**93.06**	–	**93.95**

**Figure 7:**
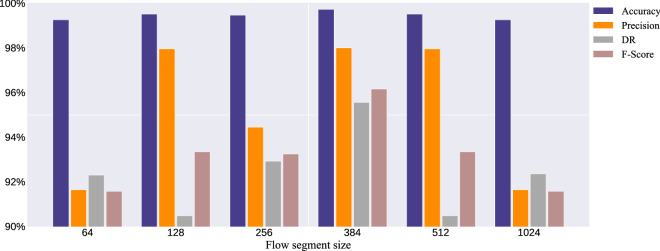
Influence of flow segment size on the proposed model.

This paper also carried out the influence of flow segment length on the HAGRU model, therefore, the length of the flow segment length $L$ is 64, 128, 256, 512, and 1024 for comparison. The experimental results are shown in Fig. 7. After considering the comprehensive indexes of “Precision”, “Detection Rate”, “FPR” and “*F*-score”, when the flow segment length is 384, the proposed HGRU model performs well. Therefore, in the experiment, the size of the novel model flow segments $L$ is 384.

Aiming at the problem of detection efficiency in malicious traffic detection, this paper proposes a data preprocessing method that uses artificial feature engineering to reduce the dimension of feature vectors. At the same time, the attack frequency is used to sample the data, and then the data flow is divided into segments to improve the detection efficiency of the model and the data throughput per unit time. ${Seq}_{i}$ (flow segment) is input to the model data element, which is composed of *L* data flow and the feature vector dimension of each data flow is *f*. The data flow segment ${Seq}_{i}$ is a continuous multiple data flow truncated by length *L*, so each data flow segment may have three situations. From a practical point of view, in a general network, the amount of normal traffic is greater than the amount of malicious traffic, and the network traffic is treated as a data flow segment, which can allow normal traffic to pass the detection quickly and only need to pay attention to the data of the malicious flow. Therefore, a malicious traffic detection model with a hierarchical attention mechanism is proposed to detect this kind of data. By fusing the feature information of the attention mechanism hierarchy, the maximum pooling hierarchy, and the average pooling hierarchy, the detection ability of the model is improved. The introduction of the attention mechanism is very important. When the model detects a large number of data flow segments, it focuses on capturing the malicious flow in the data flow segment, which can perform better in detecting malicious traffic with limited computing resources. Under the use of this advantage, the test performance of the HAGRU model proposed in this paper on the NSL-KDD, CIC-IDS2017, and CES-CIC-IDS2018 datasets are superior to other comparison models.

## Discussion

Through comparative experimental analysis, the proposed HAGRU model performs very well in classification on the total sample. And when the dataset is bigger, the data category is more, it has a certain advantage. If you look at a single category of data, the proposed model can do a good job of identifying categories with fewer data, compared with the traditional models, the proposed model can consider more types of network attacks. The main reason is that HAGRU uses attentional mechanism and hierarchies, rich features can be extracted from even a small sample of data, it can do good traffic identification in the case of data imbalance.

Although the proposed HAGRU model has advantages over other models in categories with small sample size, However, the HAGRU model could not meet the evaluation indexes of other categories with a large sample size. The reason is that this is a common problem, whether using machine learning or deep learning, the problem caused by unbalanced data categories cannot be completely solved. Because the model is based on the learning of data, the model will be biased towards the categories with a large amount of data, which will make it difficult to identify the samples with a small amount of data. However, there may be another reason for this problem. The traffic generated on different types of attacks is not always consistent with the way the time flows. In this paper, the HAGRU model is proposed as a neural network of time series traffic. For some attacks, the data traffic generated is not a time series, so the recognition effect of this kind of attack is not as good as that of other attacks. For example, on the NSL-KDD dataset, the proposed HAGRU model exceeded 98% of the *F*-score in the categories of Normal, DoS, Probe, but only 87.86% of the *F*-score in the category of U2R. This is caused not only by the small number of data samples but also by the type of U2R attack.
